# Transcriptional Profiling of Whole Blood Identifies a Unique 5-Gene Signature for Myelofibrosis and Imminent Myelofibrosis Transformation

**DOI:** 10.1371/journal.pone.0085567

**Published:** 2014-01-13

**Authors:** Hans Carl Hasselbalch, Vibe Skov, Thomas Stauffer Larsen, Mads Thomassen, Caroline Hasselbalch Riley, Morten K. Jensen, Ole Weis Bjerrum, Torben A. Kruse

**Affiliations:** 1 Department of Hematology, Roskilde Hospital, University of Copenhagen, Roskilde, Denmark; 2 Department of Clinical Genetics, Odense University Hospital, Odense, Denmark; 3 Department of Hematology X, Odense University Hospital, Odense, Denmark; 4 Department of Hematology L, Herlev Hospital, University of Copenhagen, Herlev, Denmark; 5 Department of Hematology L, Rigshospitalet, University of Copenhagen, Copenhagen, Denmark; University of Massachusetts Medical, United States of America

## Abstract

Identifying a distinct gene signature for myelofibrosis may yield novel information of the genes, which are responsible for progression of essential thrombocythemia and polycythemia vera towards myelofibrosis. We aimed at identifying a simple gene signature – composed of a few genes - which were selectively and highly deregulated in myelofibrosis patients. Gene expression microarray studies have been performed on whole blood from 69 patients with myeloproliferative neoplasms. Amongst the top-20 of the most upregulated genes in PMF compared to controls, we identified 5 genes (DEFA4, ELA2, OLFM4, CTSG, and AZU1), which were highly significantly deregulated in PMF only. None of these genes were significantly regulated in ET and PV patients. However, hierarchical cluster analysis showed that these genes were also highly expressed in a subset of patients with ET (n = 1) and PV (n = 4) transforming towards myelofibrosis and/or being featured by an aggressive phenotype. We have identified a simple 5-gene signature, which is uniquely and highly significantly deregulated in patients in transitional stages of ET and PV towards myelofibrosis and in patients with PMF only. Some of these genes are considered to be responsible for the derangement of bone marrow stroma in myelofibrosis. Accordingly, this gene-signature may reflect key processes in the pathogenesis and pathophysiology of myelofibrosis development.

## Introduction

The Philadelphia-negative chronic myeloproliferative neoplasms (MPNs) – essential thrombocythemia (ET), polycythemia vera (PV) and primary myelofibrosis (PMF) are closely related acquired stem cell neoplasms characterized by transitions between the diseases implying a biological continuum from ET over PV to the advanced myelofibrosis phase – either categorized as PMF or myelofibrosis following ET and PV [1]. These transformations occur steadily over years and are in general diagnosed by the findings of increasing splenomegaly in concert with the development of leukoerytroblastic anemia with immature circulating myeloid cells, including elevated CD34+ counts in peripheral blood [2], [3]. Several genes have been found differentially expressed in myelofibrosis, but none have been described to be selectively deregulated in this patient group [4]–[16]. Identifying a distinct gene signature for myelofibrosis may yield novel information of the genes, which are responsible for progression of the MPNs towards the burnt-out phase with dense collagenisation of the bone marrow and huge splenomegaly – a disease phase which in most patients is refractory to conventional therapy.

Using transcriptional profiling of whole blood from patients with ET, PV and PMF, we aimed at identifying a simple gene signature – composed of a few genes - which were selectively and highly deregulated in patients with myelofibrosis.

## Patients and Methods

The study was approved by The Regional Scientific Ethical Committees for Southern Denmark and was performed in accordance with the Helsinki Declaration. All patients provided written informed consent to participate in the study.

Gene expression microarray studies have been performed on whole blood from control subjects (n = 21) and patients with ET (n = 19), PV (n = 41), and PMF (n = 9). The patients were diagnosed and followed in two institutions. Most patients were studied on cytoreductive therapy, which for the large majority included hydroxyurea. Samples were collected in Paxgene tubes (Preanalytix, Switzerland) and stored for 24 hours at room temperature, then at −20°C for minimum one day, and finally transferred to a −80°C freezer. Total RNA was extracted from whole-blood using the Paxgene Blood RNA Kit (Qiagen, Franklin Lakes, NJ, USA). Quantity of RNA was determined with the NanoDrop spectrophotometer ND-8000 (NanoDrop Technologies, Wilmington, DE, USA) and quality of RNA was assessed using the Agilent 2100 Bioanalyser (Agilent Technologies, Palo Alto, CA). 300 ng of total RNA was converted to amplified RNA (aRNA) and fragmented using the MessageAmpTM III RNA amplification kit (Ambion, Austin, TX, USA). Fragmented aRNA was hybridized to Affymetrix HG-U133 Plus 2.0 chips recognizing 54,675 probe sets (38,500 genes).

The affy package (www.bioconductor.org), implemented in the statistical programming language R [17], was applied for initial data analysis. The robust multi-array average expression measure (rma) [18] was applied to perform background correction, normalization, and expression index calculation of all microarrays. Only perfect match probes were used for data analysis. The regularized t-test limma was applied to calculate differences in gene expression between patients with ET vs. controls, PV vs. controls, and PMF vs. controls [19]. Correction for multiple hypothesis testing was done using the Benjamini Hochberg method [20] and a false discovery rate (FDR) <0.05 was considered significant. Hierarchical cluster analysis was performed to reveal the expression pattern of the 5 genes in PMF compared to ET and PV. All expression values for each gene across all samples were standardized before clustering to have mean 0 and standard deviation 1. Clinical and biochemical data for ET, PV, and PMF in the clusters are reported in Table 1. To identify a potential relationship between expression values and clinical variables in patients with ET, PV, and PMF, we performed Pearson correlation analysis using SPSS 21. Data are available from Gene Expression Omnibus (http://www.ncbi.nlm.nih.gov/geo; accession no. GSE26049).

**Table 1:** Clinical and Biochemical Data in Cluster 1–5 with low, low-intermediate, intermediate, intermediate-high, and high expression values of the 5 genes, respectively.

Diagnosis	Number of patients	JAK2 V617F (+/−)	JAK2 V617F (qPCR)*	Hb-Conc. (mmol/L)*	Leukocyte Count (x 109/L)*	Neutrophile Count (x 109/L)*	Monocyte Count (x 10^9^/L)*	Thrombocyte Count (x 10^9^/L)*	Disease Duration Months*	Survival (years)**	Alive (number)**
Cluster 1											
ET	7	4/3	3.5 (0.1–24)	8.1 (7.0–9.1)	4.2 (3.9–7)	2.3 (1.87–4.13)	0.38 (0.25–0.57)	304 (218–434)	40 (12–115)	7.5 (6–14)	4 (1 unknown)
PV	11	10/1	35 (2–58)	8 (7–9.6)	5.9 (3–9)	3.13 (1.59–6.5)	0.33 (0.14–0.89)	306 (184–1030)	27 (2–171)	7 (6–20)	7 (2 unknown)
PMF	0										
All	18	14/4	28 (0.1–58)	8.1 (7–9.6)	4.2 (3–9)	2.76 (1.59–6.5)	0.34 (0.14–0.89)	305 (184–1030)	27 (2–171)	7 (6–20)	11 (3 unknown)
Cluster 2											
ET	8	4/4	19 (0.3–25)	7.9 (6.3–8.9)	6.3 (4.2–9.8)	4.1 (1.81–6.32)	0.53 (0.35–0.78)	475 (259–706)	35 (15–149)	8 (6–18)	7
PV	15	15/0	28 (5–85)	8.4 (7.2–9.4)	7.4 (3.1–10.4)	5.11 (1.61–8.42)	0.44 (0.21–1.47)	383 (202–622)	55 (12–171)	9 (4–20)	11
PMF	1	1/0	7	7.3	13.3	7.71	2.39	38	16	2	0
All	24	20/4	24 (0.3–85)	8.2 (6.3–9.4)	7.4 (3.1–13.3)	4.99 (1.61–8.42)	0.53 (0.21–2.39)	394 (38–706)	38 (12–171)	9 (2–20)	18
Cluster 3											
ET	3	2/1	29 (0.1–58)	9 (8.9–9.9)	8.4 (4.4–9.3)	6.14 (2.73–6.38)	0.42 (0.37–0.48)	516 (401–520)	38 (27–49)	11 (8–13)	3
PV	11	11/0	37 (0.2–95)	8.7 (7.3–9.9)	9.9 (5.4–17)	7.55 (3.56–14.3)	0.51 (0.19–0.83)	468 (164–581)	65 (8–163)	12 (7–19)	9
PMF	2	0/2	0	6.5 (6–6.9)	4.8 (2.2–7.3)	2.6 (0.9–4.31)	0.5 (0.2–1)	137 (42–232)	48 (11–85)	9.5 (6–13)	0
All	16	13/3	37 (0.1–95)	8.8 (6–9.9)	8.3 (2.2–17)	5.9 (0.9–14.3)	0.49 (0.19–0.83)	441 (42–581)	46 (8–163)	11.5 (6–19)	12
Cluster 4											
ET	1	1/0	67	9	15.2	14.3	0.3	268	278	26	0
PV	4	4/0	87 (29–92)	8.2 (7.8–8.7)	11.7 (10.3–15.9)	9.1 (7.79–13.8)	0.1 (0.02–0.17)	381 (220–535)	23 (13–61)	6 (2–11)	1 (1 unknown)
PMF	2	0/2	0	5.9 (5.8–6)	2.5 (1.6–3.3)	1.8 (0.98–2.57)	0.64 (0.24–1.38)	25 (19–31)	113 (22–204)	13 (8–18)	1
All	7	5/2	82 (29–92)	7.8 (5.8–9)	11.5 (1.6–15.9)	8.05 (0.98–14.3)	0.27 (0.02–1.38)	268(19–535)	23 (13–278)	9.5 (2–26)	2 (1 unknown)
Cluster 5											
ET	0										
PV	0										
PMF	4	1/3	59	7.1 (6.6–7.5)	15.3 (6–55.6)	7.9 (3.9–10.4)	1.3 (0.18–22.2)	184 (80–329)	34 (13–156)	4 (3–17)	1
All	4	1/3	59	7.1 (6.6–7.5)	15.3 (6–55.6)	7.9 (3.9–10.4)	1.3 (0.18–22.2)	184 (80–329)	34 (13–156)	4 (3–17)	1

Median and range are shown. Abbreviations: ET =  Essential Thrombocythemia; PV =  Polycythemia Vera; PMF =  Primary Myelofibrosis; *  =  blood tests at the time of blood sampling for gene expression profiling; disease duration at the time of blood sampling; **:from diagnosis as assessed January 2013.

## Results

**Figure 1:**
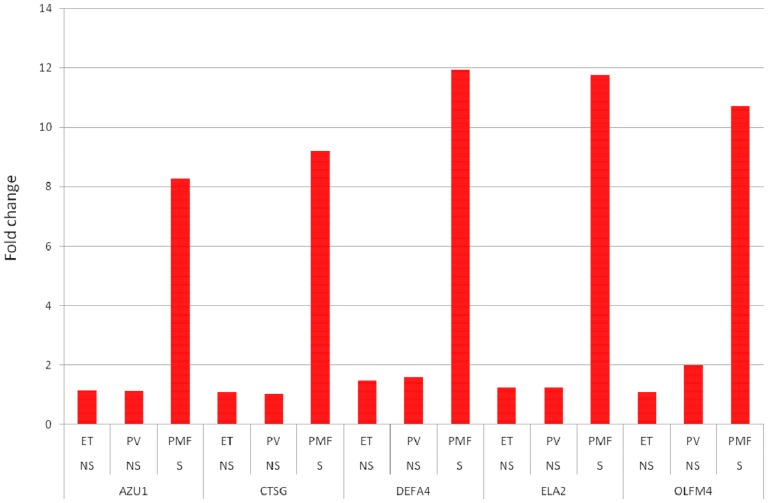
Fold changes for the 5 genes in ET, PV, and PMF compared to control subjects. Patient groups and genes are shown on the x-axis and fold changes on the Y-axis. NS: non-significant; S: significant. All genes FDR<0.05.

20,439, 25,307, and 17,417 probe sets were identified to be differentially expressed between controls and patients with ET, PV, and PMF, respectively (FDR<0.05). Amongst the top-20 most up-regulated genes in PMF, we identified 5 genes (DEFA4 (rank no. 7), ELA2 (rank no. 8), OLFM4 (rank. no. 11), CTSG (rank no. 16), and AZU1 (rank no. 18), which were uniquely and highly significantly deregulated only in PMF and not in ET and PV (FDR<0.05). Fold changes for the 5 genes in ET, PV, and PMF compared to control subjects are shown in Figure 1. Subsequent inspection of individual samples revealed that these genes were also highly expressed in 1 patient with ET and 4 patients with PV having an aggressive phenotype and/or were transforming towards myelofibrosis.

### Description of clusters

**Figure 2:**
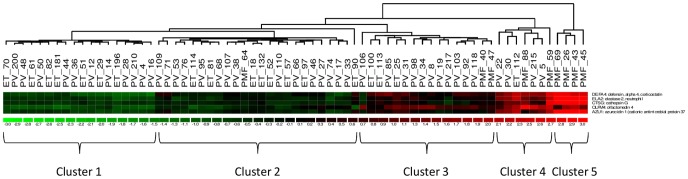
Hierarchical Cluster analysis with euclidean distance in ET, PV and PMF patients. Rows in the heat map represent the five genes DEFA4, ELA2, CTSG, OLFM4, and AZU1, and columns represent patients. The color key ranges from green to red representing standardized expression values of −3.0 to 3.0. Green indicates low expression, black intermediate expression, and red high expression. Five major clusters can be identified. Cluster 1 (green, low expression), cluster 2 (green-black, low-intermediate expression), cluster 3 (black-red, intermediate expression), cluster 4 (red-black, intermediate-high expression), and cluster 5 (red, high expression).The dendogram shows the degree of similarity between patients.

The unsupervised hierarchical cluster analysis demonstrated a distribution of patients with ET, PV, and PMF in five separate clusters (Figure 2).

#### Cluster 1

Eighteen patients (7 ET, 11 PV) were located in this cluster, all with low expression values. Two patients with ET died 7 and 8 years after diagnosis of lung cancer, respectively. One PV patient died 7 years from diagnosis of acute leukemia, and one PV patient died of splenic abscess (after infarction?) 6 years from diagnosis with no signs of transformation.

#### Cluster 2

Twenty four patients (8 ET, 15 PV, 1 PMF) were located in cluster 2, all with low-intermediate expression values. The single PMF patient in this cluster was classified as early prefibrotic MF at the time of diagnosis (JAK2-positive with 7% mutated alleles) and died of septicemia 2 years after diagnosis. The ET patient no. 18 suffered from severe comorbidity with congestive heart failure and chronic nephropathy. The ET patient no. 90 patient died 9 years after diagnosis of disseminated lung cancer. The PV patient no. 95 died of bowel cancer 5 years from diagnosis. The PV patient no. 68 had disseminated prostate cancer diagnosed 7 years from diagnosis, PV patient no. 46 transformed to AML, and PV patient no. 27 died of bowel cancer. The PV patient no. 74 had rheumatoid arthritis and died of lung cancer.

#### Cluster 3

Sixteen patients (3 ET, 11 PV, 2 PMF) were located in cluster 3 (intermediate expression). Due to the development of anemia the “ET”-patient no 25 had a new bone marrow biopsy in January 2013 (5 years from blood sampling for gene expression studies), displaying a hypercellular bone marrow with a marked increase in large megakaryocytes and also an increased endothelial proliferation with dilated sinusoids. The reticulin was increased (grade 1–2). Accordingly, in retrospective, this patient had likely early prefibrotic myelofibrosis although genuine ET at the time of blood sampling with later evolution of prefibrotic myelofibrosis cannot be excluded. The “ET”-patient no. 100 had a diagnosis of ET in 2003 when the JAK2V617F allele burden was 1%. In 2007 - at the time of blood sampling for gene expression profiling - the JAK2V617F-allele burden had increased to 57% but still without an increase in the hematocrit on treatment with hydroxyurea. In 2013 the plasma ferritin was decreased to 11 ug/L with a normal hematocrit, which – together with the increase in JAK2V61F allele burden above 50% - were considered supportive of incipient transformation to PV. The patient no. 113 had a diagnosis of “ET” in 2002, when a chromosomal analysis was normal. In 2007 the initial bone marrow biopsy was revised to early prefibrotic PMF and in October 2012 a bone marrow biopsy concluded PMF (reticulin fibrosis grade 1) with 10% myeloblasts. The patient is being considered for a stem cell transplantation. The 2 PMF patients in this cluster (no. 40 and no. 47) were JAK-negative and clustered mainly together with PV patients. Patient no. 40 was classified as early prefibrotic myelofibrosis. Patient No. 47 was diagnosed with hypercellular myelofibrosis 7 years before blood sampling for gene expression profiling and died 6 years later in deep pancytopenia.

#### Cluster 4

Seven patients (4 PV patients 1 ET and 2 PMF patients) were in this cluster (intermediate - high expression). Interestingly, in this cluster, two of the four PV patients were in the transitional stage (with 82% and 92% mutated JAK2 alleles) and two had 29% and 92% JAK2 mutated alleles, respectively. Patient No. 215 had a large tumor burden with pancytosis and massive splenomegaly from the time of diagnosis. The PV patient no. 22 had highly proliferative PV with pancytosis, hematocrit 0.75, and splenomegaly at the time of diagnosis. The patient died 2 years after diagnosis with cancer of unknown origin. The PV patient no. 30 had an initial bone marrow biopsy displaying panmyelosis with reticulin fibrosis grade 3 and - in addition - large splenomegaly and leukoerythroblastosis compatible with PMF. The patient died of pneumonia and interstitial lung fibrosis 6 years from diagnosis. Postmortem examination showed extramedullary hematopoiesis in the spleen and liver. The PV patient no. 5 had highly proliferative PV with frequent phlebotomies, leukocytosis and thrombocytosis despite treatment with hydroxyurea. The PMF patient no. 59 (JAK2 negative) – located adjacent to cluster 5 - with high expression of the 5 genes had high risk MF, weekly transfusion dependency and was subsequently cured by a minitransplantation. The PMF patient no. 88 developed myelofibrosis with pancytopenia 10 years after a diagnosis of “ET” in 1990 (likely prefibrotic myelofibrosis); the patient died 18 years from diagnosis due to septicemia. Patient No. 112 was diagnosed with ET 23 years prior to blood sampling. Two years later the patient transformed to PV and died soon after. The patient had 67% mutated JAK2 alleles compatible with transformation of ET to PV.

#### Cluster 5

Four patients were in this distinct cluster, all having myelofibrosis (3 PMF and 1 post-ET myelofibrosis). One of the PMF patients was diagnosed with ET in 1994 and transformed into classical post-ET MF with huge splenomegaly and severe bone marrow fibrosis in 2000. The patient died in leukemic transformation 2011. Another PMF patient was at the time of diagnosis highly proliferative with leukocyte count 69 MIA/L; platelet count 819 MIA/L and LDH 1084 U/L. This patient died in leukemic transformation 4 years from diagnosis. One PMF patient had a diagnosis of PMF in 2006 and from 2010 massive splenomegaly which steadily decreased during treatment with Jakavi. One PMF patient was in leukemic transformation 3 years from diagnosis and died with multi-organ failure after intensive chemotherapy.

In all patients, the expression values of the five genes correlated significantly with the leukocyte count (DEFA4: r = 0.40, p = 0.00076; ELA2: r = 0.45; p = 0.00009; CTSG: r = 0.36, p = 0.002; OLFM4: r = 0.29, p = 0.015; AZU1: r = 0.48, p = 0.00002) and neutrophil counts (DEFA4 r = 0.44; p = 0.00015; ELA2: r = 0.45,p = 0.00009; CTSG: r = 0.26,p = 0.027; OLFM4: r = 0.47, p = 0.000045; AZU1: r = 0.40, p = 0.0005). All 5 genes correlated significantly with the JAK2V617F mutation levels as well (DEFA4: r = 0.49, p = 0.00018; ELA2: r = 0.46, p = 0.00042; CTSG: r = 0.34, p = 0.01; OLFM4: r = 0.48, p = 0.00025; AZU1: r = 0.40, p = 0.0025). A significant correlation with the monocyte counts were recorded for the ELA2, CTSG and AZU1 genes only (ELA2: r = 0.27, p = 0.026; CTSG: r = 0.25, p = 0.044; AZU1: r = 0.35, p = 0.0036). A significant negative correlation with the platelet count was found for the following genes: ELA2: r = 0.27, p = 0.025; CTSG: r = 0.32, p = 0.0045; AZU1: r = 0.26, p = 0.031. When analyzing only the JAK2V617F-positive patients all five genes except for CTSG also displayed significant correlations with the leukocyte count (DEFA4: r = 0.48, p = 0.00024; ELA2: r = 0.40; p = 0.0013;; OLFM4: r = 0.53, p = 0.0000035; AZU1: r = 0.36, p = 0.0067) and the neutrophil count (DEFA4 r = 0.53; p = 0.00004; ELA2: r = 0.48,p = 0.0002; OLFM4: r = 0.61, p = 0.000001; AZU1: r = 0.43, p = 0.0013).

## Discussion

We have identified a simple 5-gene signature, which is uniquely and highly significantly deregulated in patients with PMF and in a subset of ET and PV patients, being featured by an aggressive clinical phenotype and/or a transitional stage towards myelofibrosis. The results from the hierarchical cluster analysis indicate that PMF patients with an advanced disease stage indeed have the highest expression of the five genes. Interestingly, all 3 “ET” patients in cluster 3 being initially categorized as “ET” and accordingly also having this diagnosis at the time of investigation were later reclassified to prefibrotic MF, displayed a biochemical profile suggestive of PV (an increase in allele burden >50% and low plasma ferritin despite normal hematocrit) or transformed into early prefibrotic myelofibrosis, respectively. Notably, 2 PV patients in the transitional stage clustered adjacent to the group of PMF patients with high expression values suggesting a correlation between progression of the disease to the advanced myelofibrosis stage and increasing expression of the five genes.

It was expected that the PMF patients were defined by the gene signature because the 5 genes were found by comparing control subjects with PMF patients. Indeed, these 5 genes were highly significantly deregulated even after correction for multiple hypothesis testing showing that the study had sufficiently strong power even though only 9 patients with PMF were included. Our findings that this 5-gene signature was able to capture patients with ET or PV in transitional stage or being featured by an aggressive phenotype are highly interesting, adding further support to the robustness of our gene signature in delineating this subset of patients.

All genes are encoding proteins which are constituents of neutrophil granules [21]–[24].Accordingly, this gene-signature may reflect key processes in the pathogenesis and pathophysiology of myelofibrosis development. In this context, it is intriguing to consider if these deregulated genes are responsible for the derangement of bone marrow stroma with defective cell adhesive properties due to increased proteolytic bone marrow activity and ultimately escape of immature myeloid progenitors from bone marrow niches [25], [26].

The mobilization of CD34+cells from the bone marrow into the peripheral blood is considered to be consequent to increased proteolytic activity with altered cell adherence to bone marrow stroma [25], [26]. Accordingly, the escape of CD34+ cells with dissemination and seeding extramedullarily, in particular in the spleen and liver, may be compared to the metastatic behaviour of solid tumors [27]. In the context of defective cell adhesive properties, several proteins have been described to be involved [25], the common denominator being that the large majority are constituents of neutrophil granules [21]–[24] Thus, plasma levels of neutrophil elastase and of metalloproteinase (MMP-9) have been found to be increased in PMF and PV patients with the highest values being recorded in patients with PMF [25]. In this context, it is of considerable interest to note that we found significant correlations between the five genes and both the leukocyte and neutrophil counts. Furthermore, a significant correlation was recorded between each of the five genes and the JAK2V617F-allele burden. Taking into account that the constitutive mobilization of CD34+ cells into the peripheral blood in myelofibrosis has been suggested to be due to action of a number of proteases [25] and the JAK2V617F mutation may be deeply involved in this process as well by constitutively activating granulocytes and by this means mobilizing CD34(+) cells [28] our findings are certainly supportive. In regard to MMP-9 this gene was not amongst the top-20 of the most up-regulated genes in PMF (MMP-9 was slightly upregulated in our myelofibrosis patients but not in ET and PV patients; data not shown).

Olfactomedin 4 (OLFM4) is a member of the olfactomedin-related glycoprotein family, which is specifically expressed in neutrophils and the gastrointestinal tract [29], [30]. It plays an important role in myeloid leukemia cellular functions [31] and may be a novel target gene for retinoic acids and the demethylation agent, 5-aza-2′-deoxycytidine [31]. Most recently, by analyzing the expression of OLFM4 mRNA in myeloid cells from normal human bone marrow, it has been demonstrated that OLFM4 mRNA is a genuine constituent of neutrophil specific granules [32]. Since OLFM4 is highly expressed in immature neutrophils in the bone marrow and markedly downregulated in neutrophils in peripheral blood, OLFM4 might be involved in trafficking of neutrophils from bone marrow into peripheral blood. The highly upregulated OLFM4 may also contribute to clonal expansion both by enhancing myeloproliferation and myeloaccumulation consequent to decreased apoptosis.

An alternative hypothesis on the mechanism underlying progenitor cell trafficking in MPNs implies that interactions between SDF-1α and its receptor CXCR4 are involved in the regulation of both the retention and the mobilization of haematopoietic progenitors [33]. Thus, reduced CXCR4 expression has been reported on circulating CD34+ cells from patients with myelofibrosis. In addition, the mRNA levels of CXCR4 in purified CD34+ cells from patients with MMM has been found to be lower than those from healthy subjects [34]. Taken together, the above findings suggest an altered expression and a potential signaling defect of CXCR4 to be involved in the mobilization of progenitor cells in patients with myelofibrosis.

We have recently reported a number of genes to be highly significantly deregulated in patients with MPNs, when using whole blood transcriptional profiling instead of gene expression profiling of isolated cells from bone marrow or peripheral blood (CD34+ cells, mononuclear cells, granulocytes) [12]–[16]. In addition to confirming results from single cell lineage studies or studies of mononuclear cells only - in our design very often with much higher and significant gene expression values -, our whole blood transcriptional studies have unravelled significant deregulation of additional genes adding novel information on the pathogenesis of MPNs, including the significance of chronic inflammation as a potential driver of clonal evolution in MPNs [35]–[38]. Accordingly, our studies have convincingly shown that transcriptional profiling of whole blood – the “tumor tissue” – being composed of both clonal and reactive polyclonal cells similar to other tissues being investigated by gene expression profiling – yields unique gene signatures depicting deregulation of several genes of major pathogenetic importance for MPN progression. The genes reported highly deregulated in our myelofibrosis patients confirm the robustness of our design to identify distinct genes, in this study encoding proteins which are known constituents of neutrophil granules but some of which recently demonstrated to be of significance for egress of CD34+ cells from bone marrow to peripheral blood. In conclusion, we have for the first time described a gene signature, which –in our design –seems to be unique in identifying patients with myelofibrosis and ET and PV patients with an aggressive phenotype and/or being in the transformation phase towards myelofibrosis. Studies are in progress to investigate if a similar distinct gene signature may also distinguish early prefibrotic myelofibrosis from genuine ET and in a larger series of PV-patients also may identify those who are transforming into myelofibrosis.
